# Intraventricular haemorrhage in a Ugandan cohort of low birth weight neonates: the IVHU study

**DOI:** 10.1186/s12887-020-02464-4

**Published:** 2021-01-06

**Authors:** R. MacLeod, J. N. Paulson, N. Okalany, F. Okello, L. Acom, J. Ikiror, F. M. Cowan, C. J. Tann, L. E. Dyet, C. F. Hagmann, K. Burgoine

**Affiliations:** 1 Neonatal Unit, Mbale Regional Referral Hospital, P.O. Box 1966, Mbale, Uganda; 2 Department of Biostatistics, Product Development, Genentech, Inc., South San Francisco, California USA; 3 Varimetrics Group Limited, P. O Box 2190, Mbale, Uganda; 4 Department of Paediatrics, Imperial College London, London, UK; 5 Department of Infectious Disease Epidemiology, London School of Hygiene & Tropical Medicine, Keppel Street, London, UK; 6 MRC/UVRI & LSHTM Uganda Research Unit, PO Box 149, Entebbe, Uganda; 7 Neonatal Medicine, University College London Hospitals NHS Trust, 235 Euston Road, London, UK; 8 Department of Neonatology and Pediatric Intensive Care, Children’s University Hospital of Zurich, Zurich, Switzerland; 9 Children’s Research Center, University Children’s Hospital Zurich, Zurich, Switzerland

**Keywords:** Intraventricular haemorrhage, Preterm, Low birth weight, Neonate, Low income country

## Abstract

**Background:**

Globally, 15 million neonates are born prematurely every year, over half in low income countries (LICs). Premature and low birth weight neonates have a higher risk of intraventricular haemorrhage (IVH). There are minimal data regarding IVH in sub-Saharan Africa. This study aimed to examine the incidence, severity and timing of and modifiable risk factors for IVH amongst low-birth-weight neonates in Uganda.

**Methods:**

This is a prospective cohort study of neonates with birthweights of ≤2000 g admitted to a neonatal unit (NU) in a regional referral hospital in eastern Uganda. Maternal data were collected from interviews and medical records. Neonates had cranial ultrasound (cUS) scans on the day of recruitment and days 3, 7 and 28 after birth. Risk factors were tabulated and are presented alongside odds ratios (ORs) and adjusted odds ratios (aORs) for IVH incidence. Outcomes included incidence, timing and severity of IVH and 28-day survival.

**Results:**

Overall, 120 neonates were recruited. IVH was reported in 34.2% of neonates; 19.2% had low grade (Papile grades 1–2) and 15% had high grade (Papile grades 3–4). Almost all IVH (90.2%) occurred by day 7, including 88.9% of high grade IVH. Of those with known outcomes, 70.4% (81/115) were alive on day 28 and survival was not associated with IVH. We found that vaginal delivery, gestational age (GA) < 32 weeks and resuscitation in the NU increased the odds of IVH. Of the 6 neonates who received 2 doses of antenatal steroids, none had IVH.

**Conclusion:**

In this resource limited NU in eastern Uganda, more than a third of neonates born weighing ≤2000 g had an IVH and the majority of these occurred by day 7. We found that vaginal birth, earlier gestation and need for resuscitation after admission to the NU increased the risk of IVH. This study had a high rate of SGA neonates and the risk factors and relationship of these factors with IVH in this setting needs further investigation. The role of antenatal steroids in the prevention of IVH in LICs also needs urgent exploration.

**Supplementary Information:**

The online version contains supplementary material available at 10.1186/s12887-020-02464-4.

## Introduction

Globally, 15 million neonates are born prematurely every year, with more than half being in low-income countries (LICs) [1, 2]. With rising rates of preterm birth and decreasing mortality due to improved neonatal care, the numbers of preterm neonates surviving in LICs are increasing [1, 3]. Preterm and low birth weight (LBW) neonates are at increased risk of intracranial bleeding, specifically intraventricular haemorrhage (IVH), with those < 32 weeks gestational age (GA) or < 1500 g at highest risk [4, 5]. In high income countries (HICs), IVH has been shown to contribute significantly to both mortality and cognitive and motor neurological impairment in premature and LBW neonates [4, 6–8]. However, data regarding the incidence, risk factors and complications of IVH in LICs are scarce [9–12]. This study on IVH is one of the first studies in LBW neonates in sub-Saharan Africa and the first in east Africa.

IVH is often divided into low and high grades, neurodevelopmental outcomes being different between these two groupings. Low grade IVH is defined as bleeding confined to the germinal matrix (Papile grade 1) or into the lateral ventricle without causing dilatation (Papile grade 2) [5]. High grade IVH is bleeding into the lateral ventricle causing acute ventricular dilatation (Papile grade 3) or IVH associated with periventricular haemorrhagic infarction (PVHI) (Papile grade 4) [5].

In HICs, the prevalence of IVH in neonates born weighing < 1500 g is reported at 32%, with 16% of these being high grade [13]. For neonates weighing up to 2200 g, 23% have IVH, 10.3% high grade [14]. When assessed by GA, of those born at < 28 weeks GA, 32–33.9% have IVH, 12.6–16% severe [13, 15, 16]. Neonates born at < 32 weeks have reported IVH prevalence of 21.4–24.2, 7.7% high grade [14, 17, 18]. In HICs, high grade IVH leads to cerebral palsy in about 30% of cases, mainly but not only related to the site of parenchymal haemorrhagic infarction, in addition to other developmental and neurosensory impairments [16].

In HICs, many risk factors for IVH have been identified, including lack of antenatal maternal corticosteroids, early sepsis, hypoglycaemia, respiratory distress syndrome (RDS), need for continuous positive airway pressure (CPAP), lack of postnatal vitamin K administration, hypothermia, male sex, maternal human immunodeficiency virus (HIV) infection and patent ductus arteriosus (PDA) [14, 17, 19–21]. Minimising these risk factors can reduce the incidence of IVH, hence it is important to identify the most influential and modifiable risk factors in the population in question.

Evidence from HICs is not always generalisable to LICs due to variations in population, perinatal exposures and neonatal care. Thus the contribution of IVH to death and disability in LICs warrants further investigation [1]. This study aimed to examine the incidence, severity and timing of IVH amongst LBW neonates in Uganda and examine the impact of IVH on neonatal survival. In addition, we aimed to identify modifiable risk factors for IVH to inform the future development of prevention strategies.

## Methods

### Study design

This was a prospective cohort study of neonates with birthweights of ≤2000 g who were admitted to the neonatal unit (NU) at Mbale Regional Referral Hospital (MRRH) in eastern Uganda.

### Setting

MRRH serves a mixed urban and rural population of 4.5 million people. It has a dedicated NU that admits over 2500 neonates a year, including around 600 neonates weighing ≤2000 g. Neonates are admitted from the labour ward, surrounding health facilities and home. MRRH-NU saw significant reduction in preterm mortality following implementation of a two-tiered hospital-based neonatal care package [3]. Further reduction in preterm mortality was achieved with the introduction of bubble CPAP (bCPAP) [22]. The Ugandan National Guidelines recommend antenatal steroids are given to all women in threatened preterm or preterm labour and intrapartum antibiotics are given to women with signs of sepsis, chorioamnionitis or preterm premature rupture of membranes (PPROM) but the practice is not ubiquitous [23]. Vitamin K is available for administration at delivery in MRRH and in the NU for those born elsewhere who did not receive it. Neonates of < 27 weeks GA receive palliative care. In the absence of adequate laboratory investigations, all neonates < 2000 g receive 7 days of postnatal antibiotics.

### Participants

All neonates admitted to the NU weighing ≤2000 g between January and May 2019 were screened for eligibility. Parents or guardians were approached for informed, written consent for enrolment. Inclusion criteria were i) admission weight ≤ 2000 g, ii) < 72 h old at recruitment iii) mother or guardian who spoke English, Luganda, Lumasaba or Ateso iv) mother or guardian able to provide informed, written consent and v) infant alive at the time of the recruitment scan. Exclusions included neonates with major congenital anomalies and neonates from multiple births where a sibling weighed > 2000 g.

### Data sources and measurement

On admission, heart rate and oxygen saturations were recorded using a Masimo™ Radical 7 pulse oximeter and respiratory rate was measured manually. Axillary temperature was measured using an Omron™ digital thermometer at admission and four times per day thereafter. Neonates were weighed using Seca™ electronic scales. GA was assessed using the New Ballard assessment [24]. Maternal medical history and demographics, antenatal events and birth history were collected via a structured interview with the mother and from obstetric medical records at the point of recruitment. Neonatal data regarding exposure to postnatal risk factors for IVH and clinical outcomes were extracted from medical records up to day 28 or the time of death.

The following definitions were used:

- Respiratory distress: tachypnoea, sub-costal recessions and nasal flaring within the first 4 h after birth.
- Haemodynamically significant PDA (hsPDA): confirmed on echocardiogram and defined by the presence of left atrium/ aortic root diameter > 1.5 in the presence of continuous left to right shunting.

All neonates underwent a cranial ultrasound scan (cUS) on the day of recruitment (< 72 h of age) and additional scans on days three, seven and 28. Neonates who were recruited on day three due to late presentation to the unit or delivery outside of recruitment hours had additional scans only on days seven and 28. If neonates were discharged before day 28, they were called back for their cUS. If the patient was reported to have died after discharge, the date was confirmed by the parent or guardian.

All scans were performed using a portable ultrasound machine (Sonosite M-Turbo®) with a C11x curved probe (frequency 8–5 MHz) and a linear probe (frequency 13–6 MHz). Standard images were taken with the curved probe via the anterior fontanelle in coronal and sagittal planes with additional images taken via the mastoid and posterior fontanelles [25]. The linear probe was used to obtain colour Doppler images of venous sinuses. Scans were performed by two clinical research fellows and the lead neonatal doctor, all of whom were trained either by United Kingdom trained consultant neonatologists or the MRRH lead neonatal doctor. Scans were anonymised at the point of acquisition, stored in a password protected cloud storage system, as was all data, and centrally read by two consultant neonatologists with extensive experience in preterm neuroimaging. Assessors were blinded to all clinical details of the neonate and images from each infant were grouped together. Inter observer reliability between the assessors was > 90%.

The images were examined for the presence and severity of IVH, post-haemorrhagic ventricular dilatation (PHVD), cystic periventricular leukomalacia (cPVL) [25], venous sinus thrombosis, cerebellar haemorrhage (CBH), cysts, pseudocysts, calcifications, lenticulostriate vasculopathy (LSV) and anatomical abnormalities. Papile Grade 1–2 was classified as low grade IVH, grade 3–4 as high grade [5].

### Exposures and outcomes

Exposures included delivery type, resuscitation at delivery, resuscitation during admission, hypoxia (oxygen saturations < 90%) at presentation, hypothermia in the first 24 h of admission (temperature < 36 °C), hsPDA, respiratory distress, bCPAP, being SGA (<10th centile of weight for GA [26]), antenatal corticosteroids, outborn, multiple pregnancy and maternal HIV. Outcomes included presence, severity, timing and complications of IVH including PHVD and cPVL and neonatal mortality.

### Statistical methods

A convenience sample of 120 neonates was recruited. All statistical analyses were performed using the statistical language R version 3.6.2 [27]. IVH and other ultrasound findings were reported as a proportion (%) of neonates affected. Risk factors were tabulated and presented in frequencies and percentages. For Tables 4, 5 and Supplementary Table 1, unadjusted, adjusted odds ratios (aOR) and 95% confidence intervals were calculated using logistic regression. The outcome of interest was an IVH event and risk factors were the covariates of interest. The aOR were estimated by adjusting for gestation and weight as continuous measures and sex as a binary measure. Supplementary Table 2 presents unadjusted, adjusted odds ratios and 95% confidence intervals on survival outcomes as a function of bleeds within all neonates. Separately, odds ratios (OR) were calculated when stratifying patients by low weight and gestational status. When stratifying by weight and GA the respective variables accounting for weight and GA were not included. For rare risk factors containing zero cells in the 2 × 2 contingency table we applied Fishers’ exact test to calculate unadjusted OR and 95% confidence intervals.

## Results

**Fig. 1:**
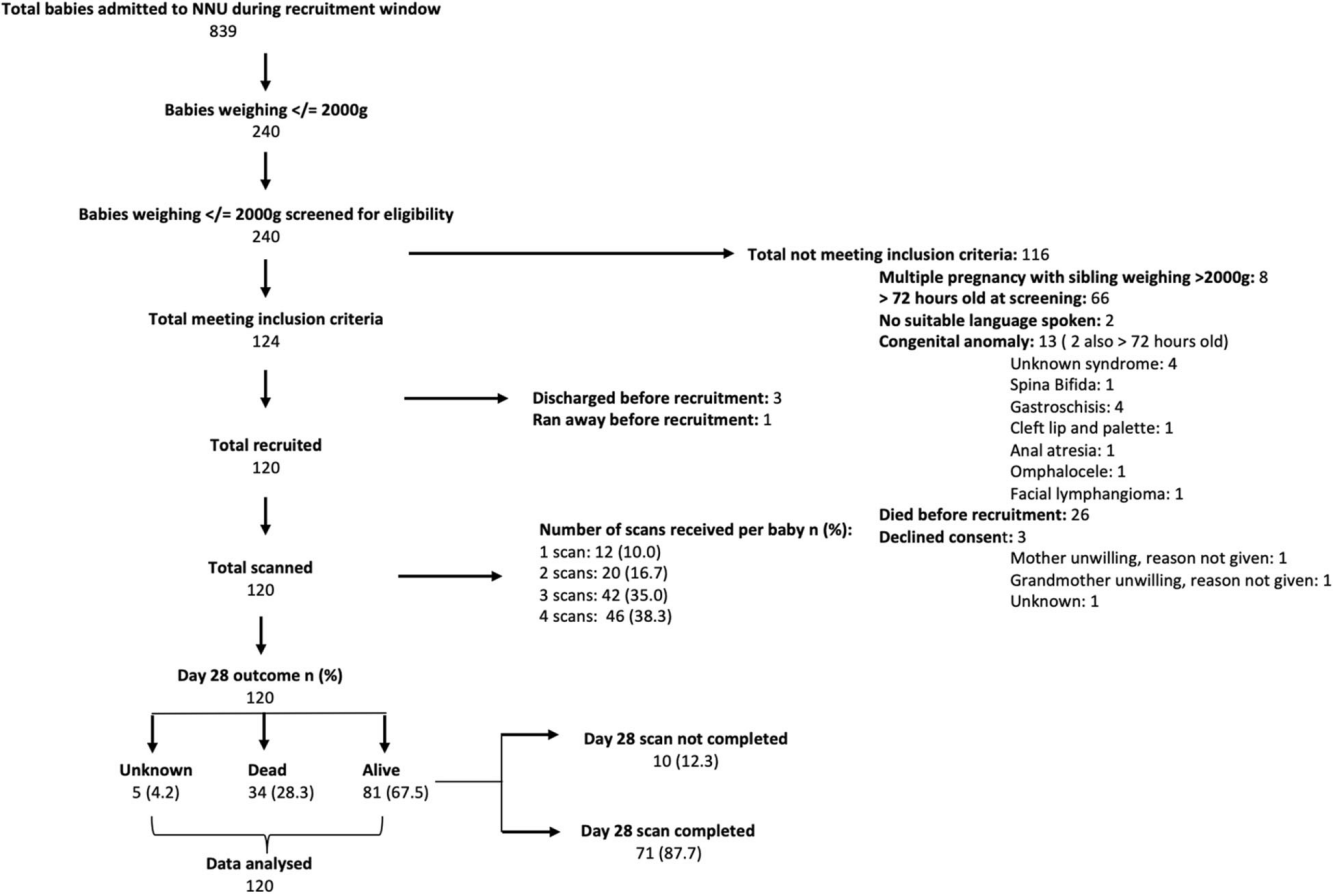
Flow chart of recruitment process, exclusions and outcomes

During the 5-month study period, 839 neonates were admitted to MRRH-NU, 240 (28.6%) of whom weighed ≤2000 g and were screened for enrolment eligibility. Inclusion criteria were fulfilled by 124 neonates and 120 were successfully recruited (Fig. 1). Table 1 shows their baseline characteristics.

**Table 1:** Patient characteristics (n, %) *N* = 120

***Sex***	
Male	60 (50)
Female	60 (50)
***Admission weight (grams)***	
< 1000	9 (7.5)
1000–1499	53 (44.2)
1500–2000	58 (48.3)
Mean (± SD)	1463 ± 310
***Gestation (weeks) (New Ballard assessment)***	
*< 28*	3 (2.5)
28–31	38 (31.6)
32–36	70 (58.3)
37–42	6 (5)
Unknown	3 (2.5)
***Age at admission (days)***	mean ± SD 1.15 ± 0.36
***SGA***	
Yes	51 (42.5)
No	66 (55)
Unknown	3 (2.5)
***Multiple pregnancy***	
Singleton	73 (60.8)
Twin	44 (36.7)
Triplet	3 (2.5)
***Delivery Type***	
Vaginal Cephalic	93 (77.5)
Vaginal Breech	5 (4.2)
Emergency Caesarean	22 (18.3)
Elective Caesarean	0 (0)
Instrumental	0 (0)
**Place of delivery**	
MRRH	64 (53.3)
District Hospital	10 (8.3)
Health Centre	27 (22.5)
Private Clinic	7 (5.8)
Home	9 (7.5)
On the way to hospital	3 (2.5)
**Antenatal steroids**	
None	61 (50.8)
1 dose	48 (40.0)
2 doses	6 (5)
Unknown	5 (4.2)
**Age at scan**	n, (Median, range)
Day 1	88 (2, 1–3)
Day 3	106 (3, 2–5)
Day 7	97 (7, 6–10)
Day 28	71 (33, 24–87)

### Incidence, severity and timing of IVH

Overall 34.2% (41/120) neonates had IVH of any severity, with 15% (18/120) having a high grade IVH (Table 2). Table 2 describes the severity of IVH seen according to birth weight and gestational age. High grade IVH was more common amongst neonates of lower gestational age and birth weight.

**Table 2:** Frequency, severity of intraventricular haemorrhage n, (%)

	Neonates ≤ 1500 g ***n*** = 62	Neonates 1500-1999 g ***n*** = 58	All neonates ≤ 2000 g ***n*** = 120	Neonates < 32 weeks ***n*** = 41^**a**^	Neonates ≥ 32 weeks ***n*** = 76^**a**^
**No haemorrhage**	38 (61.3)	41 (70.7)	79 (65.8)	27 (65.9)	50 (65.8)
**Any haemorrhage**	24 (38.7)	17 (29.3)	41 (34.2)	14 (31.6)	26 (34.1)
**Grade 1**	5 (8.1)	5 (8.6)	10 (8.3)	2 (4.9)	7 (9.2)
**Grade 2**	8 (12.9)	5 (8.6)	13 (10.8)	4 (10.4)	9 (11.8)
**Grade 3**	8 (12.9)	3 (5.2)	11 (9.2)	4 (9.8)	7 (9.2)
**Grade 4**	3 (4.8)	4 (6.9)	7 (5.8)	4 (10.5)	3 (3.9)

^a^3 neonates did not have a Ballard score recorded

**Fig. 2:**
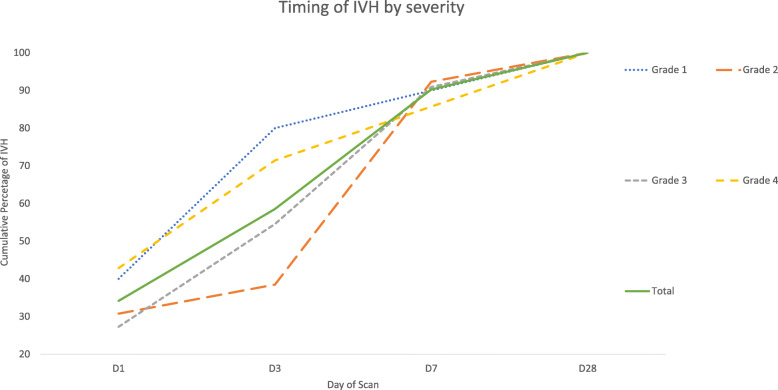
Cumulative timing of IVH by severity for all cases of IVH

Of neonates who had IVH, 90.2% had occurred by day 7, including 88.9% of all high grade IVHs (Fig. 2). Regarding grade 4 IVH (PVHI), 42.9% (3/7) occurred by day 1 and 14.3% (1/7) after day 7 (Fig. 2).

Cystic PVL (cPVL) was identified in three neonates (2.5%), one with bilateral grade 3 cPVL on day one, two on day 28, one with bilateral grade 3 cPVL and one unilateral grade 2 cPVL (Table 3). None of the cases of cPVL were associated with reported IVH. Other findings and anatomical variants are shown in Table 3.

**Table 3:** Frequency of non-IVH ultrasound findings n, (%)

Absent septum pellucidum	1 (0.8)
Calcifications	1 (0.8)
Cerebellar haemorrhage	2 (1.7)
Choroid plexus cyst	17 (14.2)
Cystic PVL Grade 2	1 (0.8)
Cystic PVL Grade 3	2 (1.7)
Lenticulostriate vasculopathy	20 (16.7)
Post haemorrhagic ventricular dilation	2 (1.7)
Pseudocysts	12 (10)
Signs of hypoxic injury	1 (0.8)
Subarachnoid haemorrhage	2 (1.7)
Venous sinus thrombosis	2 (1.7)

### Risk factors for IVH

Table 4 shows risk factors associated with IVH. Ninety-eight babies (81.7%) were born by vaginal delivery. At birth 13 (11.3%) received resuscitation including bag and mask ventilation and/or chest compressions. During admission, 10 neonates (8.3%) were resuscitated in the NU, 3 of whom had also been resuscitated at delivery. Fifty-one (42.5%) babies were SGA. The majority of mothers received no antenatal corticosteroids (55%), 40% received 1 dose and only 5% of mothers received 2 doses and (Table 4).

**Table 4:** Risk factors associated with IVH in univariable and multivariable analysis (*N* = 120)

Risk factor	Risk factor present n (%)	No IVH (***N*** = 79) n (%)	Any IVH (***N*** = 41) n (%)	Unadjusted OR (95% CI)	Adjusted OR (95% CI)
**Antepartum**					
Multiple pregnancy	47 (39.2)	34 (43.0)	13 (31.7)	0.61 (0.27–1.3)	0.54 (0.23–1.22)
Maternal HIV	7 (5.8)	3 (3.8)	4 (9.8)	2.59 (0.54–13.76)	1.90 (0.38–10.41)
Antepartum steroids (2 doses)	6 (5.0)	6 (7.6)	0 (0)	0 (0–6.19)	0 (0–1.05)
Antepartum steroids (any doses)	54 (45)	39 (49.4)	15 (36.6)	0.55 (0.25–1.20)	0.63 (0.27–1.47)
**Intrapartum**					
Vaginal delivery	98 (81.7)	60 (75.9)	38 (92.7)	4.011 (1.26–17.89)	3.50 (1.01–16.45)
Outborn	56 (46.7)	39 (49.4)	17 (41.5)	1.376 (0.65–2.98)	1.87 (0.80–4.57)
**Neonatal**					
Male	60 (50.0)	41 (52.9)	19 (346.3)	0.80 (0.37–1.70)	0.69 (0.15–3.12)
Admission weight < 1500 g^a^	62 (51.7)	38 (48.1)	24 58.5)	1.52 (0.71–3.30)	0.67 (0.15–2.93)
Gestation < 32 weeks^b^	41 (34.2)	27 (34.2)	14 (34.1)	1.00 (0.45–2.27)	6.70 (1.6–31.02)
SGA (<10th centile)	51 (42.5)	33 (41.8)	18 (43.9)	1.09 (0.50–2.36)	2.6 (0.79–9.42)
Resuscitation at delivery	13 (10.8)	11 (13.9)	2 (4.9)	0.32 (0.05–1.26)	0.36 (0.05–1.47)
Resuscitation in NU	10 (8.3)	3 (3.8)	7 (17.1)	5.22 (1.36–25.34)	5.10 (1.23–26.36)
Respiratory distress within 4 h of admission	49 (40.8)	29 (36.7)	20 (48.8)	1.64 (0.76–3.55)	1.43 (0.581–3.48)
bCPAP	38 (31.7)	20 (25.3)	18 (43.9)	2.31 (1.04–5.17)	2.09 (0.84–5.27)
hsPDA	20 (16.7)	10 (12.7)	10 (24.4)	2.23 (0.83–5.97)	1.89 (0.68–5.24)
Hypothermia (< 36 degrees) in 1st 24 h	84 (70.0)	53 (67.1)	31 (75.6)	1.46 (0.63–3.56)	1.17 (0.45–3.16)
Hypoxia (< 90% saturation in air) at presentation	47 (39.2)	31 (39.2)	16 (39.0)	0.99 (0.45–2.15)	1.00 (0.43–2.33)

^a^Adjusted for sex and gestational age, ^b^adjusted for sex and weight. All other variables adjusted for sex, gestational age and weight

Tables 4, 5 and Supplementary Table 1 report crude and adjusted ORs examining the association between IVH and a range of perinatal exposures. Prior to adjustment, resuscitation in the NU, vaginal delivery and use of bCPAP were shown to increase the odds of IVH. After adjustment for sex, weight and GA as described above, vaginal delivery, GA < 32 weeks, resuscitation in the NU and being SGA (<10th centile) were found to increase the odds of having an IVH. The aOR for having any IVH was 3.5 (95% CI 1.01–16.45), comparing vaginal delivery with Caesarean delivery. Compared with neonates of ≥32 weeks GA, neonates of GA < 32 weeks had increased odds of any IVH, aOR 6.70 (95% CI 1.6–31.02), high grade IVH, aOR 8.18 (95% CI 1.18–69.37), and low grade IVH, aOR 6.70 (95% CI 1.12–46.9). Neonates who required resuscitation in the NU also had increased odds of any IVH, aOR 5.10 (95% CI 1.23–26.36) and high grade IVH aOR 9.24 (95% CI 1.83–54.38). Neonates who were SGA had increased odds of low grade IVH, aOR 9.96 (95% CI 1.83–71.84). Birth weight < 1500 g, sex, use of bCPAP, hsPDA, respiratory distress, resuscitation at delivery, hypoxia at presentation, hypothermia in the first 24 h after birth, multiple pregnancy, maternal HIV and not being born at the study site were not shown to be associated with IVH after adjustment. When considering neonates whose mothers received any antenatal steroids, no relationship with IVH was demonstrated. Of interest, no IVH of any severity was seen in the subgroup of six infants whose mothers received 2 doses of steroids (see supplementary Table 3).

**Table 5:** Risk factors associated with high grade IVH in univariable and multivariable analysis (*N* = 97)

Risk factor	Risk factor present n (%)	No IVH (N = 79) n (%)	High grade IVH (***N*** = 18) n (%)	Unadjusted OR (95% CI)	Adjusted OR (95% CI)
**Antepartum**					
Multiple pregnancy	39 (40.2)	34 (43.0)	5 (27.8)	0.51 (0.15–1.49)	0.41 (0.12–1.27)
Maternal HIV	6 (6.2)	3 (3.8)	3 (16.7)	4.67 (0.80–27.46)	3.08 (0.48–19.38)
Antepartum steroids (2 doses)	6 (6.2)	6 (7.6)	0	0 (0–3.58)	0 (0–4.51)
Antepartum steroids (any doses)	46 (47.4)	39 (49.4)	7 (38.9)	0.59 (0.20–1.66)	0.835 (0.26–2.66)
**Intrapartum**					
Vaginal delivery	78 (80.4)	60 (75.9)	18 (100.0)	6.15 (0.86–271.26)	–
Outborn	46 (47.4)	39 (49.4)	7 (38.9)	1.53 (0.55–4.55)	3.01 (0.92–11.03)
**Neonatal**					
Male	51 (52.6)	41 (52.9)	10 (55.6)	1.16 (0.41–3.33)	1.73 (0.21–15.49)
Admission weight < 1500 g^a^	49 (50.5)	38 (48.1)	11 (61.1)	1.70 (0.61–5.03)	2.25 (0.26–20.59)
Gestation < 32 weeks^b^	35 (36.1)	27 (34.2)	8 (44.4)	0.68 (0.24–1.96)	8.18 (1.18–69.37)
SGA (<10th centile)	37 (38.1)	33 (41.8)	4 (22.2)	0.38 (0.10–1.18)	0.81 (0.14–4.36)
Resuscitation at delivery	13 (13)	11 (13.9)	2 (11.1)	0.77 (0.11–3.26)	1.19 (0.16–5.72)
Resuscitation in NU	8 (8.2)	3 (3.8)	5 (27.8)	9.74 (2.14–52.4)	9.24 (1.83–54.38)
Respiratory distress within 4 h of admission	39 (40.2)	29 (36.7)	10 (55.6)	2.16 (0.77–6.24)	1.78 (0.52–6.16)
bCPAP	29 (29.9)	20 (25.3)	9 (50.0)	2.95 (1.92–8.60)	2.28 (0.67–7.77)
hsPDA	15 (15.5)	10 (12.7)	5 (27.8)	2.65 (0.73–8.86)	2.36 (0.62–8.45)
Hypothermia (< 36 degrees) in 1st 24 h	69 (71.1)	53 (67.1)	16 (88.9)	3.77 (0.97–25.03)	3.84 (0.87–27.67)
Hypoxia (< 90% saturation in air) at presentation	41 (42.3)	31 (39.2)	10 (55.6)	2.12 (0.74–6.41)	2.01 (0.64–6.51)

^a^ Adjusted for sex and gestational age, ^b^ adjusted for sex and weight. All other variables adjusted for sex, gestational age and weight

### Outcomes at 28 days

Of the 120 neonates recruited, 34 (28.3%) died before day 28. Twenty-eight (23.3%) died before discharge, including 16 (13.3%) before day 7. An additional six (5%) died after discharge. The majority, 79.4% (27/34), of neonates who died weighed < 1500 g at birth. Five neonates (4.2%) were lost to follow-up and 81 (70.4%) were seen and had a day 28 cUS (Fig. 1). IVH was not found to be an independent risk factor for death, even if severe (Supplementary Table 2).

## Discussion

This is one of the first studies of IVH in LBW neonates in a LIC in sub-Saharan Africa and the first in east Africa. We found that more than a third of neonates born weighing ≤2000 g had any IVH and 15% had high grade IVH. The majority of IVH had occurred by day 7. Vaginal delivery, GA < 32 weeks, resuscitation in the NU and being SGA (<10th centile) increased the odds of having IVH.

Our findings show a higher prevalence of IVH than two studies from Nigeria, but are comparable to one study from Zambia. This prospective study of neonates weighing < 1500 g found an IVH prevalence of 34.2%, comparable to our finding of 38.7% [10]. Neonates were only scanned up to day 7 however, and our study showed 9.8% of IVH occurred after day 7. In contrast, the studies from Nigeria, both of neonates < 1500 g found lower rates of IVH. One found a 24.1% prevalence of IVH, however each infant was only scanned once within the first week (range 60 h to 7 days) [9]. The second found 29.7% had IVH, 7.5% of which were high grade [12]. This is likely to be an underestimation as many eligible neonates were not scanned as they died within 72 h of birth. No studies in LICs in sub-Saharan Africa have examined IVH in neonates weighing 1500-2000 g. Our data showing that in this subgroup almost one in three have IVH and one in 10 have high grade IVH are therefore the first in this area.

On analysis by gestation, in neonates of 28–31 weeks GA we found rates of IVH of 31.6%, comparable with findings from another Nigerian study of 33.3% [11]. However, in the 32–42 week GA group we found much higher rates (34.2% vs 6.3%). Of note, this study had much lower rates of SGA neonates than ours (21.8% vs 42.5%) which may contribute to this difference, although the relationship of SGA with IVH varies between studies [28, 29].

Overall and in all subgroups for any IVH and high grade IVH, our rates are higher than in HICs. The difference is smallest in the < 1500 g and < 28 week gestation groups and largest in neonates of > 1500 g or > 37 weeks, suggesting in our setting the higher weight and GA neonates may benefit most from modification of risk factors [13, 14, 16, 18, 30].

Our results show that 34% of all IVH had occurred on day 1, 58.5% by day 3 and 90.2% by day 7. In the Zambian study, 68.7% of IVH had occurred by 72 h of age, however as they only scanned up to day 7 this may be an overestimation of the total proportion [10]. Evidence from HICs shows that approximately 50% of IVHs occur in the first 24 h after birth and almost all by 72 h [31]. This could be due to different aetiologies for IVH in our settings, with more influence from postnatal risk factors due to the standard of neonatal care available.

Our results show that neonates with GA of < 32 weeks had increased odds of having both mild and severe IVH, in keeping with other studies from HICs and LICs [9–11, 13, 20, 32]. Birthweight was not shown to be associated with IVH, which is contrary to most findings from HICs but in keeping with some studies in Africa [5, 11, 12, 19]. This lack of association of weight with IVH is possibly due to the high prevalence of SGA neonates (42.5%) giving a higher GA than expected for birthweight, thus comparatively reducing risk of IVH.

Vaginal delivery was associated with increased odds of IVH compared with Caesarean delivery. It was not shown to be associated with low grade IVH, however the sample was too small to determine the relationship with high grade IVH. Whilst vaginal delivery is a recognised risk factor for high grade IVH in HICs, this finding has not been replicated in studies from LICs in sub-Saharan Africa [9–11, 33, 34]. This may be related to the timing and indications for Caesarean differing.

Resuscitation in the NU was associated with an increased probability of any IVH and high grade IVH. Resuscitation at birth is a risk factor for IVH however this was not demonstrated in our results [35]. This is likely due to poor documentation and limited accessibility of adequate neonatal resuscitation. We found that the prevalence cPVL (2.4%) was comparable with expected rates from HICs; there are no comparable data currently available from LICs in Africa [36].

We found that SGA neonates had increased odds of low grade IVH, however this finding should be interpreted with caution. Comparable studies in LICs in sub-Saharan Africa found no association with SGA and IVH, and in HICs the relationship of SGA with IVH varies between studies [11, 12, 28, 29]. Whilst our study had both a larger sample size and much higher rates of SGA than other African studies which may explain why we identified an association, this finding is not supported by any relationship with high grade IVH [11, 12]. Low grade IVH can be difficult to detect using cUS, so it is possible that some were missed in these studies [37]. Many risk factors for SGA are present in our population, including maternal malnutrition, infections and PET and their prevalence and relationship with IVH in this setting is an area of future interest [38].

Whilst no relationship between exposure to 1 or 2 doses of antenatal steroids and IVH was shown possibly due to the small sample, of the 6 neonates who received 2 doses of antenatal steroids, none had IVH. Interestingly, this group had few additional risk factors for IVH (see Supplementary Table 3). Antenatal corticosteroids are protective against IVH in HICs, however current evidence suggests that in LICs antenatal steroids can increase the risk of neonatal death [14, 39, 40]. Although it is possible that the low rates of steroid exposure in our study (only 47%) may have contributed to the high rates of IVH, further data on the impact of antenatal steroids on IVH and other preterm complications are needed; use of antenatal steroids in this setting remains controversial and needs further exploration.

We did not find IVH to be an independent predictor of survival. There is little evidence on survival after IVH in LICs, but in this cohort there was a much lower mortality in neonates with IVH than in a Nigerian study (41.7% vs 66.7%) [9]. This is likely due to the relatively high standard of care at our centre [22].

Over a quarter of neonates in the study died by day 28, the large majority before discharge. Of neonates ≤2000 g who had IVH, a third died, compared with a quarter of those who did not, a difference not found to be statistically significant. Of the neonates that died, more than three quarters weighed < 1500 g and of neonates weighing < 1500 g, just under half died, findings which are in keeping with data from a meta-analysis of neonatal mortality in East Africa [41].

Strengths of this study included that the cUS were performed by well-trained investigators and that image analysis was undertaken prospectively and independently by 2 blinded, experienced, senior clinicians. Outcomes are known for a high proportion of participants (115/120, 95.8%) and 71/81 (87.7%) babies alive at day 28 underwent a day 28 cUS.

Limitations include challenges with reliable capture of obstetric and perinatal data. Additionally, 26 neonates died very early before recruitment, to which IVH could have contributed, therefore the occurrence, particularly of severe IVH may be underestimated. This could also cause an underestimation in the mortality associated with IVH. Furthermore, 10/81 surviving neonates (12.3%) were unable to attend for day 28 scans so it is possible that some findings such as cPVL were underestimated. Due to the date of women’s last menstrual period being rarely known, assessment of GA was by Ballard scoring which is accurate to +/− 2 weeks and has inter user variability. The assessment of haemodynamic significant of PDA was made using only LA/Aortic root diameter as measurement of other parameters of haemodynamic significance were not available, therefore it is possible that may have been underestimated in some neonates due to offloading of the LA through a patent foramen ovale. Assessing potential risk factors for IVH was a secondary analysis and therefore the sample size may have been too small to determine relationships for some risk factors. Additionally, clinical data was collected retrospectively from the medical notes.

## Conclusion

This study found that in this resource limited NU in a regional referral hospital in eastern Uganda, more than a third of neonates born weighing ≤2000 g had an IVH and majority of these occurred by day 7. We found that vaginal delivery, GA < 32 weeks, resuscitation in the NU and being SGA (<10th centile) were associated with increased odds of having an IVH. This study had a high rate of SGA neonates and the risk factors and relationship of these with IVH in this setting needs further investigation. The role of antenatal steroids in the prevention of IVH in LICs also needs urgent exploration.

## Supplementary Information

**Additional file 1.**

**Additional file 2.**

**Additional file 3.**

## Data Availability

The datasets used and/or analysed during the current study are available from the corresponding author on reasonable request.

## Abbreviations

aOR: adjusted odds ratio
APH: Antepartum haemorrhage
bCPAP: bubble continuous positive airway pressure
CPAP: Continuous positive airway pressure
cPVL: cystic periventricular leukomalacia
cUS: cranial ultrasound scan
GA: Gestational age
HIC: High income country
HIV: Human immunodeficiency virus
hsPDA: haemodynamically significant patent ductus arteriosus
IVH: Intraventricular haemorrhage
KMC: Kangaroo mother care
LA: Left atrium
LBW: Low birth weight
LIC: Low income country
LOS: Late onset sepsis
MRRH: Mbale regional referral hospital
NEC: Necrotising enterocolitis
NU: Neonatal unit
OR: Odds ratio
PDA: Patent ductus arteriosus
PET: Pre-eclampsia
PHVD: Post haemorrhagic ventricular dilatation
pPROM: preterm prolonged rupture of membranes
PVHI: Periventricular haemorrhagic infarction
PVL: Periventricular leukomalacia
RDS: Respiratory distress syndrome
SSS: Superior sagittal sinus
SVD: Spontaneous vaginal delivery
