# Supplementary material for: Intraventricular haemorrhage in a Ugandan cohort of low birth weight neonates: the IVHU study
Source: BMC Pediatr. 2021 Jan 6;21:12. doi: 10.1186/s12887-020-02464-4 (PMC7786968; doi:10.1186/s12887-020-02464-4)
Supplement: Supplementary file 1 — Additional file 1. [file 12887_2020_2464_MOESM1_ESM.docx]

| **Table 5: Risk factors associated with low-grade IVH in univariable analysis (n = 102)** | | | | | |
| --- | --- | --- | --- | --- | --- |
| **Risk factor** | **Risk factor present**  **n (%)** | **No IVH**  **(n = 79)**  **n (%)** | **Low grade IVH (n = 23)**  **n (%)** | **Unadjusted OR**  **(95% CI)** | **Adjusted OR**  **(95% CI)** |
| **Antepartum** | | | | | |
| Multiple pregnancy | 42 (41.2) | 34 (43.0) | 8 (38.4) | 0.706 (0.258 – 1.82) | 0.64 (0.22 – 1.71) |
| Maternal HIV | 4 (3.9) | 3 (3.8) | 1 (4.3) | 1.11 (0.05 – 9.21) | 1.00 (0.05 – 8.60) |
| Antepartum steroids (2 doses) | 6 (5.9) | 6 (7.6) | 0 (0) | 0 (0 – 2.90) | 0 (0 – 2.42) |
| Antepartum steroids (any doses) | 47 (46.1) | 39 (49.4) | 8 (34.8) | 0.53 (0.19 – 1.38) | 0.47 (0.16 – 1.33) |
| **Intrapartum** | | | | | |
| Vaginal delivery | 80 (78.4) | 60 (75.9) | 20 (87.0) | 2.11 (0.63 – 9.65) | 2.21 (0.59 – 11.01) |
| Outborn | 49 (48.0) | 39 (49.4) | 10 (43.5) | 1.27 (0.50 – 3.29) | 1.27 (0.45 – 3.71) |
| **Neonatal** | | | | | |
| Male | 50 (49.0) | 41 (52.9) | 9 (39.1) | 0.60 (0.22 – 1.52) | 0.365 (0.06 – 2.22) |
| Admission weight <1500g* | 51 (50.0) | 38 (48.1) | 13 (56.5) | 1.40 (0.55 – 3.65) | 0.34 (0.05 – 1.95) |
| Gestation <32 weeks^ | 33 (32.4) | 27 (34.2) | 6 (26.1) | 1.44 (0.52 – 4.40) | 6.70 (1.12 – 46.9) |
| SGA (<10^th^ centile) | 47 (46.1) | 33 (41.8) | 14 (60.9) | 2.33 (0.89 – 6.46) | 9.96 (1.83 – 71.84) |
| Resuscitation at delivery | 11 (10.8) | 11 (13.9) | 0 (0) | 2.39 (0.19 – 22.30) | - |
| Resuscitation in NU | 5 (4.9) | 3 (3.8) | 2 (8.7) | 2.41 (0.30 – 15.49) | 2.23 (0.25 – 16.6) |
| Respiratory distress within 4 hours of admission | 39 (38.2) | 29 (36.7) | 10 (43.5) | 1.33 (0.51 – 3.40) | 1.30 (0.43 – 3.85) |
| bCPAP | 29 (28.4) | 20 (25.3) | 9 (39.1) | 1.90 (0.70 – 5.03) | 1.96 (0.64 – 5.88) |
| hsPDA | 15 (14.7) | 10 (12.7) | 5 (21.7) | 1.92 (0.54 – 6.15) | 1.76 (0.47 – 5.95) |
| Hypothermia (<36 degrees) in 1^st^ 24 hours | 68 (66.7) | 53 (67.1) | 15 (65.2) | 0.88 (0.34 – 2.45) | 0.59 (0.18 – 1.91) |
| Hypoxia (<90% saturation in air) at presentation | 37 (36.3) | 31 (39.2) | 6 (26.1) | 0.52 (0.17 – 1.42) | 0.61 (0.19 – 1.84) |

* Adjusted for sex and gestational age, ^ adjusted for sex and weight. All other variables adjusted for sex, gestational age and weight.
