# Supplementary material for: Intraventricular haemorrhage in a Ugandan cohort of low birth weight neonates: the IVHU study
Source: BMC Pediatr. 2021 Jan 6;21:12. doi: 10.1186/s12887-020-02464-4 (PMC7786968; doi:10.1186/s12887-020-02464-4)
Supplement: Supplementary file 2 — Additional file 2. [file 12887_2020_2464_MOESM2_ESM.docx]

Supplementary Table 2

| **Supplementary Table 2: Rates of IVH in all cases of neonatal deaths with the associated odds ratios for mortality** | | | | |
| --- | --- | --- | --- | --- |
| **Group** | **Died with no IVH**  **n (%)** | **Died with any IVH**  **n (%)** | **Unadjusted OR**  **(95% CI)** | **Adjusted OR**  **(95% CI)** |
| **All IVH** | | | | |
| All neonates^*^ | 21/79 (26.6) | 13/41 (31.7) | 1.31 (0.56 – 3.00) | 1.16 (0.40 – 3.24) |
| Weight <1500g^^^ | 17/38 (44.7) | 10/24 (41.7) | 0.88 (0.31 – 2.48) | 0.87 (0.27 – 2.69) |
| <32 weeks gestation^$^ | 9/27 (33.3) | 8/14 (57.1) | 2.52 (0.68 – 9.98) | 1.47 (0.27 – 7.74) |
| ≥32 weeks gestation^$^ | 10/50 (20) | 5/26 (19.2) | 1.00 (0.28 – 3.25) | 0.85 (0.19 – 3.34) |
| **High grade IVH** | | | | |
|  | **Died with no IVH**  **n (%)** | **Died with high grade IVH* n (%)** | **Unadjusted OR**  **(95% CI)** | **Adjusted OR**  **(95% CI)** |
| All neonates^*^ | 21/79 (26.6) | 6/18 (33.3) | 1.57 (0.48 – 4.79) | 1.72 (0.39 – 7.13) |
| Weight <1500g^^^ | 17/38 (44.7) | 4/11 (36.3) | 0.71 (0.16 – 2.75) | 0.57 (0.10 – 2.68) |
| <32 weeks gestation^$^ | 9/27 (33.3) | 3/8 (37.5) | 1.13 (0.20 – 5.77) | 0.75 (0.07 – 5.48) |
| ≥32 weeks gestation^$^ | 10/50 (20) | 3/10 (30.0) | 2.28 (0.41 – 11.03) | 2.06 (0.34 – 11.34) |
| **Low grade IVH** | | | | |
|  | **Died with no IVH**  **n (%)** | **Died with low grade IVH* n (%)** | **Unadjusted OR**  **(95% CI)** | **Adjusted OR**  **(95% CI)** |
| All neonates^*^ | 21/79 (26.6) | 7/23 (30.4) | 1.15 (0.39 – 3.11) | 0.94 (0.23 – 3.58) |
| Weight <1500g^^^ | 17/38 (44.7) | 6/13 (46.2) | 1.06 (0.29 – 3.78) | 1.40 (0.32 – 6.05) |
| <32 weeks gestation^$^ | 9/27 (33.3) | 5/6 (83.3) | 9.44 (1.27 – 196.17) | 16.98 (1.56 – 768.74) |
| ≥32 weeks gestation^$^ | 10/50 (20) | 2/16 (12.5) | 0.54 (0.08 – 2.40) | 0.48 (0.07 – 2.22) |

* Adjusted for sex, weight and gestation, ^ adjusted for sex and gestation, § adjusted for sex and weight.
