# Supplementary material for: Intraventricular haemorrhage in a Ugandan cohort of low birth weight neonates: the IVHU study
Source: BMC Pediatr. 2021 Jan 6;21:12. doi: 10.1186/s12887-020-02464-4 (PMC7786968; doi:10.1186/s12887-020-02464-4)
Supplement: Supplementary file 3 — Additional file 3. [file 12887_2020_2464_MOESM3_ESM.docx]

| **Supplementary Table 3: Demographics of all neonates receiving 2 doses of antenatal steroids.** | | | | | | | | | | | | |
| --- | --- | --- | --- | --- | --- | --- | --- | --- | --- | --- | --- | --- |
| **Neonate** | **GA (weeks)** | **BW (g)** | **Sex** | **Place of delivery** | **SGA <10^th^ centile** | **Resuscitation at delivery** | **Delivery type** | **Respiratory**  **Distress** | **CPAP** | **Apnoea** | **Resuscitation in NNU** | **Outcome** |
| 1 | 34 | 850 | Male | Inborn | Yes | Yes | Emergency Caesarean Section | Yes | No | No | No | Died |
| 2 | 35 | 2000 | Male | Inborn | No | No | Emergency Caesarean Section | No | No | No | No | Alive |
| 3 | 32 | 1280 | Female | Inborn | No | No | Normal Vaginal Delivery | Yes | Yes | No | No | Died |
| 4 | 34 | 780 | Female | Inborn | Yes | No | Emergency Caesarean Section | No | No | No | No | Died |
| 5 | 33 | 1240 | Male | Inborn | No | No | Emergency Caesarean Section | Yes | No | No | No | Alive |
| 6 | 31 | 1220 | Male | Inborn | No | No | Emergency Caesarean Section | Yes | Yes | No | No | Died |
| GA: Gestational age by Ballard assessment, SGA: Small for gestational age (<10^th^ centile for GA), CPAP: any time during admission, Apnoea: ever reported during admission (regardless of need for resuscitation). | | | | | | | | | | | | |
